# Effects of Negation and Uncertainty Stratification on Text-Derived Patient Profile Similarity

**DOI:** 10.3389/fdgth.2021.781227

**Published:** 2021-12-06

**Authors:** Luke T. Slater, Andreas Karwath, Robert Hoehndorf, Georgios V. Gkoutos

**Affiliations:** ^1^Centre for Computational Biology, College of Medical and Dental Sciences, Institute of Cancer and Genomic Sciences, University of Birmingham, Birmingham, United Kingdom; ^2^Institute of Translational Medicine, University Hospitals Birmingham, NHS Foundation Trust, Birmingham, United Kingdom; ^3^University Hospitals Birmingham National Health Service Foundation Trust, Birmingham, United Kingdom; ^4^MRC Health Data Research UK (HDR UK) Midlands, Birmingham, United Kingdom; ^5^Computer, Electrical and Mathematical Sciences & Engineering Division, Computational Bioscience Research Center, King Abdullah University of Science and Technology, Thuwal, Saudi Arabia; ^6^National Institute for Health Research Experimental Cancer Medicine Centre, Birmingham, United Kingdom; ^7^National Institute for Health Research Surgical Reconstruction and Microbiology Research Centre, Birmingham, United Kingdom; ^8^National Institute for Health Research Biomedical Research Centre, Birmingham, United Kingdom

**Keywords:** semantic similarity, phenotype profiles, ontology, context disambiguation, negation, differential diagnosis

## Abstract

Semantic similarity is a useful approach for comparing patient phenotypes, and holds the potential of an effective method for exploiting text-derived phenotypes for differential diagnosis, text and document classification, and outcome prediction. While approaches for context disambiguation are commonly used in text mining applications, forming a standard component of information extraction pipelines, their effects on semantic similarity calculations have not been widely explored. In this work, we evaluate how inclusion and disclusion of negated and uncertain mentions of concepts from text-derived phenotypes affects similarity of patients, and the use of those profiles to predict diagnosis. We report on the effectiveness of these approaches and report a very small, yet significant, improvement in performance when classifying primary diagnosis over MIMIC-III patient visits.

## Introduction

Natural language text is a critical resource in healthcare, forming the primary mode of communication and source of record (1). Analysis of clinical text resources can lead to novel insights and improved patient outcomes (2). Biomedical ontologies are tightly interlinked with text mining, since they provide sets of vocabularies that can be used to recognize concepts in text, and can be linked back to consensus definitions of mentioned entities (3).

Ontologies also enable the semantic analysis of biomedical entities described by associations with ontology classes. Semantic similarity is one such method, which leverages an ontology's subsumptive hierarchical structure to calculate similarity between concepts and groups of concepts (4), controlling for ambiguity and variability in ontology-based descriptions of entities via background knowledge encoded into the structural features of the ontology. Semantic similarity has been heavily explored as a method for predicting protein-protein similarity (5), gene-disease associations (6, 7), differential diagnosis for rare diseases (8), and disease stratification and diagnosis in particular disease domains (9, 10). There are a wide range of semantic similarity and related measures, which may compare single terms, or groups of terms. Gan et al. (4) distinguishes between methods based upon measuring relatedness via semantic relatedness, hierarchical structure, term features, information content, as well as hybrid methods. Information content measures can also be distinguished between those that are calculated via structural or semantic features of the ontology, and those that are determined through an external source (such as probability of appearing in a corpus). Methods of computing distance between vector embeddings, such as via cosine similarity, also constitute a kind of semantic similarity, and more recent investigations have also combined embedding and ontology approaches for semantic analysis (11, 12).

Text-derived annotations associated with ontology concepts can also be used as entity profiles for semantic similarity analysis. Several previous works described methods that produced patient phenotype profiles using a hybrid concept recognition approach with human curation, which could then be passed to gene variant prioritization software (13, 14). Our previous work explored the use of uncurated text-derived patient phenotypes produced by the Komenti semantic text-mining framework (15), for classification of common disease classification, with a view toward using the technology as a method for differential diagnosis (16). Particularly, we used semantic similarity methods for ranking and classification of primary diagnosis across MIMIC-III patient visits, revealing a promising, albeit error-prone, method. This implies that attention should be given to optimization of methods, including the choice of which ontology classes to include in a patient's phenotype profile.

Information extraction systems typically associate algorithmically-derived metadata with extracted concept mentions for the purpose of context disambiguation, including negation, uncertainty, and temporal status. This is of importance in a clinical setting since the context with which a concept is mentioned in a clinical narrative facilitates determination of the nature of the relationship between the concept and the patient. For example, the mere mention of a disease in clinical note for a patient does not necessarily imply that the patient has that disease. It may be negated, ruling out that disease, or it may refer to the patient being tested for a disease, or it may refer to another person entirely, such as a family member.

Context disambiguation is, therefore, a major area of research in natural language processing. In this article, we will focus on negation and uncertainty detection. NegEx is a popular rule-based context detection algorithm for clinical text (17), which supports negation and uncertainty identification. More recent approaches, such as NegBERT (18), apply machine learning approaches to negation detection. Other approaches use methods based on grammatical sentences models, such as negation-detection (19). Our previous work described a heuristic-based method, integrated into Komenti, which exhibited high performance on MIMIC-III and on text associated with rare disease patients at University Hospitals Birmingham (20).

In this work we explore whether removal of text-mined patient-concept associations determined as negated or uncertain from patient phenotype profiles, affects the performance of similarity-based classification of shared primary diagnosis. To do this, we repeat the patient-patient phase of the experiment described by our previous differential diagnosis work, and compare the performance when uncertain and negated annotations are removed from the patient phenotype profile.

## Methods

**Figure 1:**
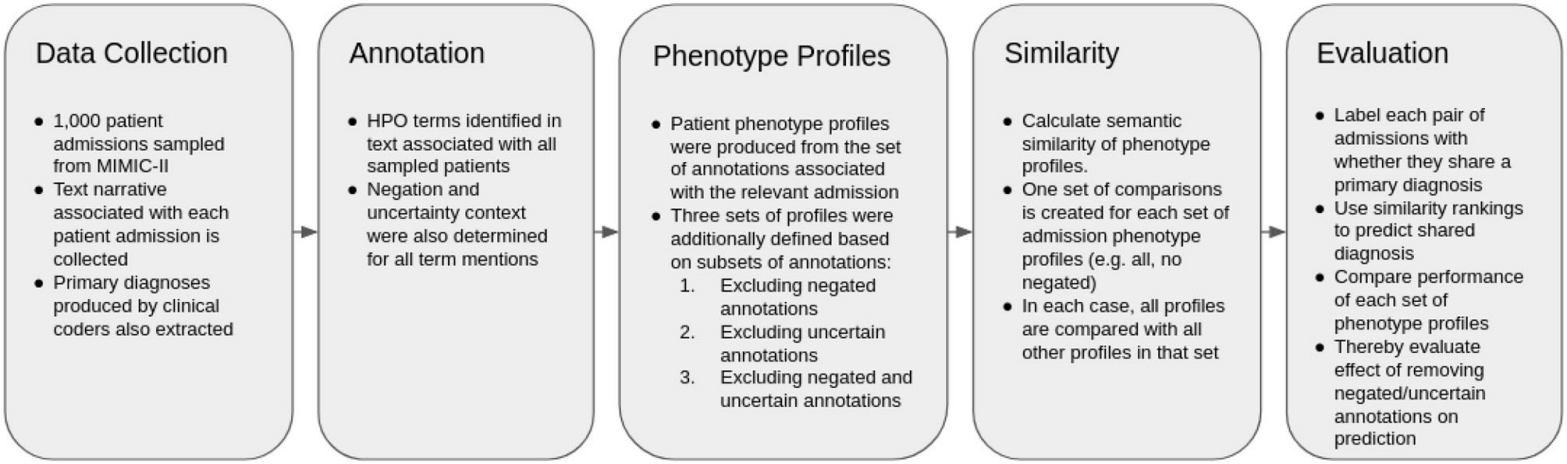
Flow chart describing the experimental methodology.

The overall methodology is described in Figure 1. The method is based heavily upon (16), which investigates the use of semantic similarity to predict shared diagnosis from phenotype profiles produced from text records in MIMIC-III. As such, the methodology is mostly equivalent. The difference is that we also only test patient-patient comparisons, and thus also do not consider any retraining in this work, and that we test the performance of different subsets of profiles, via subsetting based on results of context disambiguation of annotations.

###  Data Preparation and Information Extraction

MIMIC-III (MIMIC) is a freely available healthcare dataset, describing nearly 60,000 critical care visits across three hospitals with a combination of structured and unstructured data, including natural language text associated with patient visits (21). Within MIMIC, diagnoses are provided in the form a canonical ICD-9 code, produced in the original care setting by clinical coding experts.

We used the same set of patient visits described in our previous work (16). In the previous study, 1,000 patient visits were sampled from MIMIC, collecting their associated texts together into one file per patient visit. Patient visit sampling was limited to patients with primary diagnoses that contained an ICD-9 mapping in the Disease Ontology (DO) (22). Patients themselves were not considered as unique, which could be considered a potential source of bias, since the same individual may be hospitalized for the same or similar diseases.

We then used the Komenti semantic text mining framework (15) to create a vocabulary based on all non-obsolete terms in the Human Phenotype Ontology (HPO). Komenti is a tool that queries biomedical ontologies for text-mining vocabulary, and then implements the Stanford CoreNLP library (23) to use those vocabularies to recognize ontology terms in text. As a result of this process, sets of ontology terms describing texts can be derived. HPO is a biomedical ontology that provides formal definitions for a large range of human phenotypes (24). Subsequently, we applied the Komenti framework to annotate the texts associated with each sampled patient visit, producing a list of HPO terms associated with each patient visit, or a phenotype profile for each patient visit. Negation and uncertainty of each concept mention was determined using the komenti-negation algorithm (20), which has been tested specifically upon a MIMIC-III cohort annotated with HPO terms, exhibiting high performance upon manual validation.

The full set of patient phenotype profiles, including all annotations derived from texts associated with each patient admission, forms the baseline sample. This will subsequently be referred to as PP_*ALL*_. Then, different sets of phenotype profiles were produced by subsetting the profiles given in PP_*ALL*_, based on context disambiguation stratification for each annotation. PP_*NoNeg*_ describes the set of patient phenotype profiles with all negated annotations removed, PP_*NoUnc*_ describes the set of profiles with all uncertain annotations removed, while PP_*NoNegNoUnc*_ describes the set of profiles with all negated and all uncertain annotations removed. **Table 2** includes a list of these subsets with descriptions and annotation counts.

###  Semantic Similarity and Evaluation

We then compared every patient visit phenotype profile with every other patient visit phenotype profile, producing a ranking of similar patient visits for each patient visit. To calculate the semantic similarity scores, we used the Semantic Measures Library (25), which implements a large range of semantic comparison methods that can be used to derive measures of relatedness between ontology terms or groups of ontology terms. We applied the Resnik measure of pairwise similarity (26), which is defined as the information content of the most informative common ancestor of the classes being compared. The information content used was also defined by Resnik, defined as the negative log probability of the term appearing a corpus, where in this case the corpus was formed of all annotations making up phenotype profiles in the set currently being considered (for example, only non-negated annotations were considered in the corpus for PP_*NoNeg*_). The intent of this measure is to downregulate terms appearing frequently, and conversely upregulate terms that appear infrequently. Best Match Average was used for groupwise similarity (27), which finds, for each term in set A, the best matching term in set B. The same process is repeated in the direction of sets B and A. The average is then taken of all best matching terms, to produce the final score. This measure is intended to capture similarity of groups through several of their component terms (e.g., similarity of cardiac phenotype, and similarity of respiratory phenotype).

We then measured the ability of ranked similarity scores to be predictive of primary diagnosis. Each pairwise comparison between admission profiles was labeled with true or false, based on whether or not (respectively) those patients shared a primary diagnosis, constituting a set of “predictions” formed from the ground truth label, and both global and local similarity rankings. Global similarity rankings were ordered by the ranking of similarity scores between phenotype profiles across all pairwise rankings. Local similarity rankings were produced for each profile, ordered by ranking of similarity scores for every profile that the considered profile was compared to. The global ranking was used to produce Area Under the receiver operating characteristic Curve (AUC), while local rankings were used to produce Mean Reciprocal Rank (MRR), and Top Ten Accuracy (the percentage of profiles for whom the correct diagnosis was in the top ten most similar entities). *P*-values were calculated using the Mann-Whitney-*U* test on the ranks of patients with matching primary diagnoses compared with the baseline set of patient profiles with all phenotypes included, and we identify significance at a 0.01 cutoff. The software we created to run the experiment is freely available at https://github.com/reality/miesim.

## Results

We created phenotype profiles for 1,000 patient visits sampled from MIMIC-III, by associating them with HPO terms identified in their associated text narrative using Komenti's concept recognition features. Each annotation was also evaluated for uncertainty and negation using the komenti-negation algorithm, with the counts for each modifier summarized in Table 1.

**Table 1:** The number of annotations across the text records associated with the 1,000 sampled patients, and associated modifiers.

**Total annotations**	**Negated**	**Uncertain**	**Negated and uncertain**
43,953	8,057	3,102	317

*Each annotation was evaluated for uncertainty and negation, which are not mutually exclusive*.

We used the full set of annotations to create four sets of patient phenotype profiles, including all annotations (PP_*All*_), discluding negated annotations PP_*NoNeg*_, discluding uncertain annotations PP_*NoUnc*_, and discluding both negated and uncertain annotations PP_*NoNegNoUnc*_. These are summarized with their annotation counts in Table 2. For each of of the patient phenotype profile sets, all profiles were compared to all other profiles using semantic similarity. Using the resultant similarity matrix, we evaluated how well the ranking of similar profiles for each patient visit was predictive of shared primary diagnosis.

**Table 2:** Summary of each set of patient phenotype profiles considered as an experimental setting.

**Identifier**	**Description**	**Annotations**
PP_*All*_	All phenotypes included	43,953
PP_*NoNeg*_	Negated annotations removed	35,896
PP_*NoUnc*_	Uncertain annotations removed	40,851
PP_*NoNegNoUnc*_	Negated and uncertain annotations removed	33,111

*The phenotype profiles are formed from the list of annotations associated with each patient. Different sets were formed by removing sets of annotations depending on the contextual uncertainty and negation modifiers associated with them by Komenti*.

Table 3 summarizes the results, showing that, overall, there was a very small difference in the performance between all settings. The PP_*NoNeg*_ and PP_*NoNegNoUnc*_ profiles led to moderate increases in AUC, MRR, and A@10, with a significantly different ranking of shared diagnoses. In the PP_*NoUnc*_ setting, AUC was increased in comparison to PP_*All*_, though MRR and A@10, were reduced, with the rankings of shared diagnoses not significantly different to those in PP_*All*_.

**Table 3:** Results of classification of shared primary diagnosis, compared between different sets subsets of patient phenotype profiles.

**Setting**	**AUC**	**MRR**	**A@10**	**p-value**
PP_*All*_	0.7743 (0.7724–0.7762)	0.423	0.606	–
PP_*NoNeg*_	0.7795 (0.7776–0.7814)	**0.442**	0.615	3.588e-09
PP_*NoUnc*_	0.7804 (0.7786–0.7823)	0.421	0.599	0.4463
PP_*NoNegNoUnc*_	**0.7888 (0.7869–0.7906)**	0.437	**0.619**	3.3e-15

*A@10 refers to the percentage of patient visits whose ten most similar patient visits contained at least one patient visit sharing a primary diagnosis. P-value is calculated by Mann-Whitney-U test on rank of true matches compared to PP_All_.*

*Bold values indicate the greatest values for AUC, MRR, and A@10*.

## Discussion

While the margins are small in the cases of improved performance observed when negated, uncertain, or both, annotations were removed from phenotype profiles, the rank of correct pairings was shown to have changed significantly in the case of PP_*NoNeg*_ and PP_*NoNegNoUnc*_, while there was either no crossover in 0.95 confidence interval boundaries on AUC measures, indicating that these are statistically significant improvements (except in the case of true case rankings for PP_*NoUnc*_. Even small improvements in performance can be impactful in a clinical environment, since even the correct diagnosis of one more patient is desirable. To put the Accuracy@10 results in perspective, the PP_*NoNegNoUnc*_ set found a correct diagnosis in the top ten in 13 more cases than PP_*All*_, accounting for 1.3% of the total sample of patient visits. Since the running-time costs of Komenti's context disambiguation algorithm are small, removal of negated and uncertain annotations can therefore be seen as worthwhile.

Unlike the other reduced sets, PP_*NoUnc*_ performance is lower than PP_*All*_. It's possible that the Komenti uncertainty algorithm is unsuitable for the critical care MIMIC-III domain, as unlike the negation algorithm it relies on an uncertainty dictionary that was adapted for rare disease outpatients, and cannot fall back upon evaluating dependency resolution relation types to identify uncertainty (15). It's also possible that uncertain concept mentions are more informative and relevant to the primary diagnosis than the negated annotations, although the PP_*NoNegNoUnc*_ set indicates that there is a combined positive effect to removing both negated and uncertain annotations from phenotype profiles, implying a dependent relationship between the two, likely that removal of uncertain annotations alone enables increased information content and contribution to similarity of errant inferences drawn from negated annotations.

This brings up the greater problem that negated and uncertain annotations do actually provide additional information. For example, patients who have the same disease may be tested for the same or similar conditions, or be suspected of other diseases that have a similar presentation. Lack of certain symptoms may also be diagnostic, such as lack of pain when a patient has nerve damage or certain neurological conditions. Negative mentions may also indicate what is typical for a patient in their condition, even if that particular patient does not have that phenotype. For example, in our previous work (16), we reported a strong prevalence of pain-related discussion throughout the dataset. A patient suffering a disease that often causes pain may be asked regularly about pain, even if they deny it (causing a negative annotation). Therefore, the negated mention of pain in the clinical notes is actually relevant, and may provide useful information in identifying the patient's diagnosis.

This indicates that instead of simply discluding negated and uncertain mentions, there may be value in identifying ways to take the contextual indicators into account for similarity calculation. To our knowledge, this has not been widely explored in the context of biomedical ontologies. However, recent approaches have investigated richer use of semantic similarity, including vector-based approaches, which could potentially permit expression of more advanced expressions of the context of instances (11). It is also possible that these influences could be implanted directly into a novel comparison method, by encoding a quantitative influence into the semantic similarity measure, or by calculating separate similarity scores from affirmed, negated, and uncertain annotations, and finding a beneficial way to combine them into a groupwise measure. Such an approach could also use the quantity of negated or uncertain annotations across the corpus in the information content calculation, which could also provide benefits to phenotype associations outside of that class (e.g., strong weighting to an uncommon affirmed case, in a corpus with many negated cases).

The role of passed-on error must also be considered. Our previous work showed that the negation algorithm was very accurate in this context (20), however it and all negation algorithms evaluated involved some level of error. In addition, the performance of negation algorithms may vary wildly when applied to different datasets, depending on the application domain, adaptation performed, and transferability of the model. To some extent, this effect is mitigated by the use of all annotations in the patient phenotype profile. An important patient phenotype is likely to appear several times in a patient's associated text narrative, and the relatively low rate of error in detecting negatives means that it is unlikely that multiple instances of the phenotype are incorrectly classified (although in some cases it may be dependent upon the context with which a condition is mentioned, making multiple errors more likely).

Performance of the algorithm in general will also be affected by the clinical domain and setting. Different kinds of diseases, text data, language, coding priorities, and more will be expressed in different datasets. Thus far, the method has only been applied to MIMIC-III datasets, and thus the transferability of the approach (as well as the utility of removing negated or uncertain annotations in other settings) is untested. This speaks to the necessity of investigating the methods on other datasets and clinical settings.

Other metadata may also be helpful for optimization, such as temporal information. Indeed, like most classification approaches, the inclusion, disclusion, manner or weighting of use of certain kinds of annotation, should be treated as a process of hyper-parameter optimization. However, given the relatively few applications of the technology in the clinical space, more research must be done on the influence of these different properties, such that knowledge of effective hyperparameters can be established.

## Conclusions

We showed that exclusion of negated and uncertain annotations from text-derived patient phenotype profiles leads to a small but significant improvement in performance, when ranking patients for shared primary diagnosis with semantic similarity. We expect that these modified annotations are actually informative, but a more expressive semantic similarity method could be needed to properly leverage this information.

## Data Availability Statement

Publicly available datasets were analyzed in this study. This data can be found at: http://mimic.physionet.org/.

## Ethics Statement

Ethical review and approval was not required for the study on human participants in accordance with the local legislation and institutional requirements. Written informed consent from the participants' legal guardian/next of kin was not required to participate in this study in accordance with the national legislation and the institutional requirements.

## Author Contributions

LS conceived of the study, performed the experiments, and wrote the first draft of the manuscript. AK and RH contributed to experimental design and analysis of results. GG contributed to experimental design, manuscript development, and project supervision. All authors contributed to the article and approved the submitted version.

## Funding

GG and LS acknowledge support from support from the NIHR Birmingham ECMC, NIHR Birmingham SRMRC, Nanocommons H2020-EU (731032) and the NIHR Birmingham Biomedical Research Centre and the MRC HDR UK (HDRUK/CFC/01), an initiative funded by UK Research and Innovation, Department of Health and Social Care (England) and the devolved administrations, and leading medical research charities. RH and GG were supported by funding from King Abdullah University of Science and Technology (KAUST) Office of Sponsored Research (OSR) under Award No. URF/1/3790-01-01. AK was supported by the Medical Research Council (MR/S003991/1) and the MRC HDR UK (HDRUK/CFC/01).

## Author Disclaimer

The views expressed in this publication are those of the authors and not necessarily those of the NHS, the National Institute for Health Research, the Medical Research Council or the Department of Health.

## Conflict of Interest

The authors declare that the research was conducted in the absence of any commercial or financial relationships that could be construed as a potential conflict of interest.

## Publisher's Note

All claims expressed in this article are solely those of the authors and do not necessarily represent those of their affiliated organizations, or those of the publisher, the editors and the reviewers. Any product that may be evaluated in this article, or claim that may be made by its manufacturer, is not guaranteed or endorsed by the publisher.

## References

[1] Pereira L Rijo R Silva C Martinho R. Text mining applied to electronic medical records: a literature review. Int J E Health Med Commun. (2015) 6:1–18. 10.4018/IJEHMC.2015070101

[2] Dalianis H. Clinical Text Mining. Cham: Springer International Publishing (2018).

[3] Spasic I Ananiadou S McNaught J Kumar A. Text mining and ontologies in biomedicine: making sense of raw text. Brief Bioinformatics. (2005) 6:239–51. 10.1093/bib/6.3.239 16212772

[4] Gan M Dou X Jiang R. From ontology to semantic similarity: calculation of ontology-based semantic similarity. Sci World J. (2013) 2013:793091. 10.1155/2013/793091 23533360 PMC3603583

[5] Zhang SB Tang QR. Protein–protein interaction inference based on semantic similarity of gene ontology terms. J Theor Biol. (2016) 401:30–7. 10.1016/j.jtbi.2016.04.020 27117309

[6] Hoehndorf R Schofield PN Gkoutos GV. Analysis of the human diseasome using phenotype similarity between common, genetic and infectious diseases. Sci Rep. (2015) 5:10888. 10.1038/srep10888 26051359 PMC4458913

[7] Smedley D Oellrich A Köhler S Ruef B Sanger Mouse Genetics Project Westerfield M. PhenoDigm: analyzing curated annotations to associate animal models with human diseases. Database. (2013) 2013:bat025. 10.1093/database/bat025 23660285 PMC3649640

[8] Köhler S Schulz MH Krawitz P Bauer S Dölken S Ott CE. Clinical diagnostics in human genetics with semantic similarity searches in ontologies. Am J Hum Genet. (2009) 85:457–64. 10.1016/j.ajhg.2009.09.003 19800049 PMC2756558

[9] Paul R Groza T Zankl A Hunter J. Semantic similarity-driven decision support in the skeletal dysplasia domain. In: Cudré-Mauroux P, Heflin J, Sirin E, Tudorache T, Euzenat J, Hauswirth M, et al., editors. The Semantic Web – ISWC 2012. Lecture Notes in Computer Science. Berlin; Heidelberg: Springer (2012). p. 164–79.

[10] Steichen O Bozec CDL Thieu M Zapletal E Jaulent MC. Computation of semantic similarity within an ontology of breast pathology to assist inter-observer consensus. Comput. Biol. Med. (2006) 36:768–88. 10.1016/j.compbiomed.2005.04.014 16197935

[11] Kulmanov M Smaili FZ Gao X Hoehndorf R. Semantic similarity and machine learning with ontologies. Brief Bioinformatics. (2021) 22:bbaa199. 10.1093/bib/bbaa199 33049044 PMC8293838

[12] Kulmanov M Smaili FZ Gao X Hoehndorf R. Machine learning with biomedical ontologies. bioRxiv. (2020) 2020.05.07.082164. 10.1101/2020.05.07.082164

[13] Liu C Peres Kury FS Li Z Ta C Wang K Weng C. Doc2Hpo: a web application for efficient and accurate HPO concept curation. Nucleic Acids Res. (2019) 47:W566–70. 10.1093/nar/gkz386 31106327 PMC6602487

[14] Son JH Xie G Yuan C Ena L Li Z Goldstein A. Deep phenotyping on electronic health records facilitates genetic diagnosis by clinical exomes. Am J Hum Genet. (2018) 103:58–73. 10.1016/j.ajhg.2018.05.010 29961570 PMC6035281

[15] Slater LT Bradlow W Hoehndorf R Motti DF Ball S Gkoutos GV. Komenti: a semantic text mining framework. bioRxiv. (2020) 2020.08.04.233049. 10.1101/2020.08.04.233049

[16] Slater LT Karwath A Williams JA Russell S Makepeace S Carberry A. Towards Similarity-Based Differential Diagnostics For Common Diseases. bioRxiv. (2021) 2021.01.26.428269. 10.1101/2021.01.26.428269 PMC8204262 33836447

[17] Chapman WW Bridewell W Hanbury P Cooper GF Buchanan BG. A simple algorithm for identifying negated findings and diseases in discharge summaries. J Biomed Inform. (2001) 34:301–10. 10.1006/jbin.2001.1029 12123149

[18] Khandelwal A Sawant S. NegBERT: a transfer learning approach for negation detection and scope resolution. arXiv [Preprint] arXiv:191104211 (2020).

[19] Gkotsis G Velupillai S Oellrich A Dean H Liakata M Dutta R. Don't let notes be misunderstood: a negation detection method for assessing risk of suicide in mental health records. In: Proceedings of the Third Workshop on Computational Linguistics and Clinical Psychology. San Diego, CA: Association for Computational Linguistics (2016). p. 95–105.

[20] Slater LT Bradlow W Motti DF Hoehndorf R Ball S Gkoutos GV. A fast, accurate, and generalisable heuristic-based negation detection algorithm for clinical text. Comput Biol Med. (2021) 130:104216. 10.1016/j.compbiomed.2021.104216 33484944 PMC7910278

[21] Johnson AEW Pollard TJ Shen L Lehman LwH Feng M Ghassemi M. MIMIC-III, a freely accessible critical care database. Sci Data. (2016) 3:1–9. 10.1038/sdata.2016.35 27219127 PMC4878278

[22] Schriml LM Arze C Nadendla S Chang YWW Mazaitis M Felix V. Disease ontology: a backbone for disease semantic integration. Nucleic Acids Res. (2012) 40:D940–6. 10.1093/nar/gkr972 22080554 PMC3245088

[23] Manning CD Surdeanu M Bauer J Finkel JR Bethard S McClosky D. The Stanford CoreNLP natural language processing toolkit. In: Proceedings of 52nd Annual Meeting of the Association for Computational Linguistics: System Demonstrations. Baltimore, MD (2014). p. 55–60.

[24] Köhler S Vasilevsky NA Engelstad M Foster E McMurry J Aymé S. The Human Phenotype Ontology in 2017. Nucleic Acids Res. (2017) 45:D865–76. 10.1093/nar/gkw1338 27899602 PMC5210535

[25] Harispe S Ranwez S Janaqi S Montmain J. The semantic measures library and toolkit: fast computation of semantic similarity and relatedness using biomedical ontologies. Bioinformatics. (2014) 30:740–2. 10.1093/bioinformatics/btt581 24108186

[26] Resnik P. Using information content to evaluate semantic similarity in a taxonomy. arXiv [Preprint] arXiv:9511007 (1995).

[27] Wang JZ Du Z Payattakool R Yu PS Chen CF. A new method to measure the semantic similarity of gO terms. Bioinformatics. (2007) 23:1274–81. 10.1093/bioinformatics/btm087 17344234

